# ^19^F-perfluorocarbon-labeled human peripheral blood mononuclear cells can be detected *in vivo* using clinical MRI parameters in a therapeutic cell setting

**DOI:** 10.1038/s41598-017-19031-0

**Published:** 2018-01-12

**Authors:** Corby Fink, Jeffrey M. Gaudet, Matthew S. Fox, Shashank Bhatt, Sowmya Viswanathan, Michael Smith, Joseph Chin, Paula J. Foster, Gregory A. Dekaban

**Affiliations:** 1 Molecular Medicine Research Laboratories, Robarts Research Institute and Department of Microbiology & Immunology, University of Western Ontario, 1151 Richmond Street North, London, Ontario N6A 5B7 Canada; 2 Imaging Research Laboratories, Robarts Research Institute and Department of Microbiology & Immunology, University of Western Ontario, 1151 Richmond Street North, London, Ontario N6A 5B7 Canada; 3 200 Elizabeth Street, University Health Network, Toronto, Ontario M5G 2C4 Canada; 4 IBBME, University of Toronto, University Health Network, 200 Elizabeth Street, Toronto, Ontario M5G 2C4 Canada; 5 Division Of Surgery, Division of Surgical Oncology, London Health Sciences Centre, 800 Commissioners Rd E, London, Ontario N6A 5W9 Canada

## Abstract

A ^19^Fluorine (^19^F) perfluorocarbon cell labeling agent, when employed with an appropriate cellular MRI protocol, allows for *in vivo* cell tracking. ^19^F cellular MRI can be used to non-invasively assess the location and persistence of cell-based cancer vaccines and other cell-based therapies. This study was designed to determine the feasibility of labeling and tracking peripheral blood mononuclear cells (PBMC), a heterogeneous cell population. Under GMP-compliant conditions human PBMC were labeled with a ^19^F-based MRI cell-labeling agent in a manner safe for autologous re-injection. Greater than 99% of PBMC labeled with the ^19^F cell-labeling agent without affecting functionality or affecting viability. The ^19^F-labeled PBMC were detected *in vivo* in a mouse model at the injection site and in a draining lymph node. A clinical cellular MR protocol was optimized for the detection of PBMC injected both at the surface of a porcine shank and at a depth of 1.2 cm, equivalent to depth of a human lymph node, using a dual ^1^H/^19^F dual switchable surface radio frequency coil. This study demonstrates it is feasible to label and track ^19^F-labeled PBMC using clinical MRI protocols. Thus, ^19^F cellular MRI represents a non-invasive imaging technique suitable to assess the effectiveness of cell-based cancer vaccines.

## Introduction

Cancer immunotherapy is an emerging research area that relies on one’s own immune system to combat the cancer. Early research focused on non-specific up-regulation of the immune system using interleukins or adjuvants in an effort to elicit an anti-tumor response^1^. More recently, specific anti-tumor immune responses have been developed using tumor antigen (Ag)-specific vaccine approaches^2,3^. An example of such an immunotherapy is a tumor-specific professional antigen presenting cell (APC)-based cancer vaccine. In order for APC, such as B cells, monocytes, macrophages, and dendritic cells (DC)^4^, to function as adjuvants in cancer vaccines, they must seed secondary lymphoid organs such as a lymph node or spleen, in which interactions with CD4^+^ and CD8^+^ T cells occur^5^.

To launch a successful anti-tumor immune response, two impediments must be overcome. First, as a tumor Ag is derived from a self-Ag, the immune system is biased towards tolerance and suppression of a tumor Ag-specific immune response. Secondly, even in the case where non-self tumor neo-antigens exist, the immunosuppressive environment established by tumors elicits defective innate and adaptive anti-tumor effector responses, which coincides with deficient APC maturation and activation. Due to these aforementioned obstacles, it is advantageous to prepare properly matured and/or activated tumor Ag-specific APC *ex vivo* and then reintroduce these cells into the patient to avoid the *in vivo* immunosuppressive effects hindering proper APC priming, maturation and activation. This *ex vivo* approach has proven safe and nontoxic^6,7^ and has led to improvements in quality of life and overall survival times^8–12^.

Previous research has shown that only 3–5% of originally injected therapeutic DC reach the lymph node post injection, which is a major factor, limiting the effectiveness of mixed APC- and DC-based cancer vaccines^13–18^. As the quantity of tumor Ag-loaded APC that reach a secondary lymphoid organ and interact with T cells is directly proportional to the ensuing tumor-Ag specific T cell response elicited *in vivo*, there is a pressing need to confirm and quantify this migration non-invasively. By tracking and quantifying therapeutic cell migration, it may be possible to predict the magnitude of the anti-tumor immune response *in vivo*. This information may serve as a surrogate marker to assess and improve upon the effectiveness of APC-based cancer vaccines. Magnetic resonance imaging (MRI) is a promising approach for tracking APC^19,20^. Various types of cell labeling agents are available for pre-clinical cell tracking by MRI including iron oxide, gadolinium, manganese and [^19^F]-fluorine^21^. However, only [^19^F]-fluorine incorporated into a perfluorocarbon nanoemulsion (^19^F-PFC) is a commercially available formulation (CS-1000) designed specifically for *in vivo* cell tracking that can stably label many cell types^22–28^. This ^19^F-PFC has been approved as an investigational new drug by the U.S. Food and Drug Administration for human use as a cellular MRI tracking agent^29^ and has been employed to track human DC in mice^30,31^ and in one human clinical trial^32^. This type of cellular MRI labeling agent is attractive because it provides a quantifiable, positive signal in the absence of any endogenous ^19^F signal following an *in vivo* ^19^F MRI scan. This allows for the unambiguous detection of greater than 10^4 19^F-PFC-labeled cells per voxel^31^. When combined with ^1^H MRI, the anatomical 3-dimensional location of this observed ^19^F signal can be determined as well^28–30^. Moreover, this type of imaging is capable of translating *in vivo* ^19^F signal into the relative number of cells at a given location^30^. This allows the direct quantification of therapeutic cell numbers that migrate to secondary lymphoid organs or a tumor post-injection, and thus, has the potential to serve as a non-invasive marker predictive of the immunological outcome of a cell-based cancer immunotherapy.

This study investigates the suitability of using a ^19^F-PFC agent as an MRI cell labeling agent for a heterogeneous APC population as found in a peripheral blood mononuclear cell (PBMC)-based cancer vaccine platform such that they are detectable *in vivo*. Successful labeling of individual leukocyte and non-leukocyte lineages has been reported^23,28,30^. However, while successful ^19^F-PFC labeling of all cells in a heterogenous population might be anticipated, it has not been successfully reported^33^. Vaccines composed of a heterogeneous mixture of leukocytes will contain both non-professional APC (T and NK cells) in addition to professional APC, such as B cells, monocytes and circulating DC. PBMC form the basis of an FDA-approved immunotherapy for prostate cancer^34,35^. This pre-clinical validation study was performed as a step towards a clinical trial. The main purpose was to determine if we could label the major blood leukocyte lineages within PBMC with the ^19^F-PFC labeling agent while maintaining viability and functionality that meets the criteria for autologous re-injection. We demonstrate that ^19^F-PFC-labeled PBMC can be detected in a mock human *in vivo* system using a clinical 3 T MRI scanner and a custom-built dual ^1^H/^19^F switchable radio frequency (RF) coil suitable for use in humans.

## Results

### Human PBMC cell lineages important for antigen presentation efficiently label with ^19^F-PFC without affecting functionality

**Figure 1:**
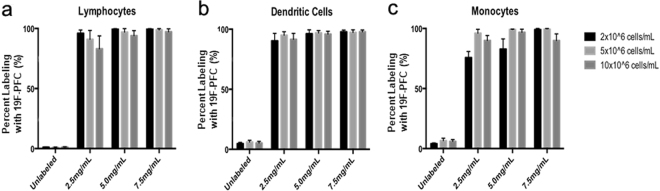
All cell lineages within human PBMC important for antigen presentation label with ^19^F-PFC. Human PBMC were cultured overnight with a red fluorescent version of the ^19^F-PFC and flow cytometry was used to qualitatively assess ^19^F-PFC incorporation. Three different cell concentrations (2–10 × 10^6^ cells/mL) and three different ^19^F-PFC labeling concentrations were employed (2.5–7.5 mg/mL). **(a**,**b**) CD3^+^ and CD19^+^ T and B cell lymphocytes (**a**) and CD11c^+^ dendritic cells (**b**) label with a high percentage regardless of cell or ^19^F-PFC concentration. (**c**) CD14^+^ monocytes label most consistently at a high percentage (>98%) when a cell concentration of 5 × 10^6^ cells/mL and a ^19^F-PFC concentration of 5 mg/mL is used (n = 3, mean ± SEM).

A range of cell (2–10 × 10^6^ cells/mL) and ^19^F-PFC (2.5–7.5 mg/mL) culture concentrations were used to determine the optimal concentrations that provide the most efficient labeling without affecting viability. It was determined that regardless of cell or ^19^F-PFC concentration, B and T cell lymphocytes, monocytes and dendritic cells all labeled with ^19^F-PFC at a high percentage (>70%, Fig. 1). Furthermore, CD14^+^ monocytes, lymphocytes and CD11c^+^CD14^−^CD16^−^ dendritic cells labeled equivalently at a high percentage when a cell concentration of 5 × 10^6^ cells/mL and 5 mg/mL ^19^F-PFC was used (Fig. 1) while maintaining a high viability at 89.47% ± 2.39% (mean ± SEM, n = 3). Therefore, the latter culture condition was chosen for all subsequent experiments presented in this report.

**Figure 2:**
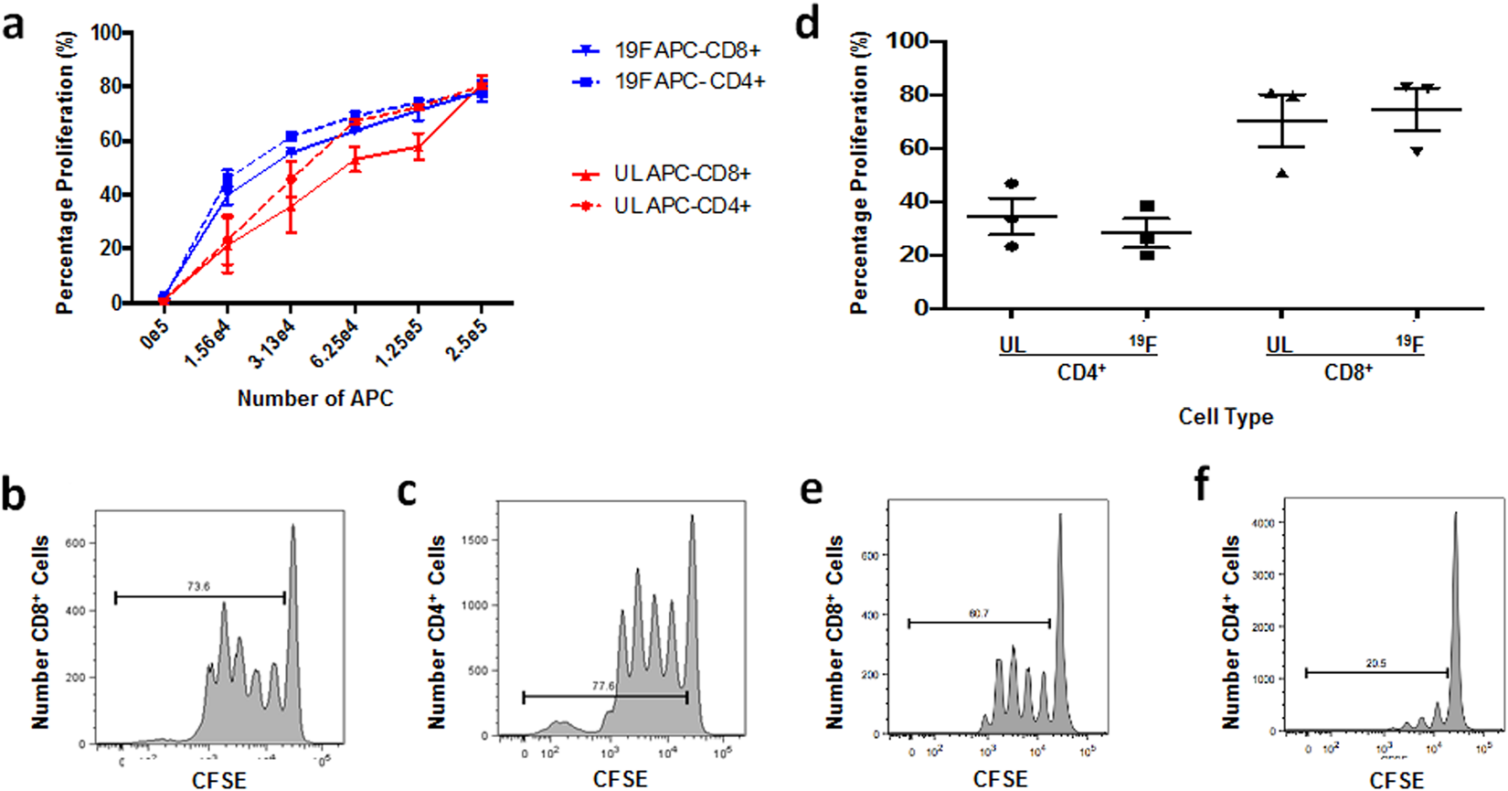
The functionality of both APC and T cells is not affected as a result of ^19^F-PFC labeling. (**a**–**c**) PBMC from healthy volunteers were either labeled with ^19^F-PFC overnight (5 mg/mL) or remained unlabeled and then subjected to T cell positive selection removal. Remaining labeled and unlabeled APC were then employed in a MLR (**a**) by co-culturing with allogeneic CFSE^+^ T cells. Flow cytometry was used to assess T cell proliferation using CFSE dilution as a readout for both CD8^+^ (**b**) and CD4^+^ T cells (**c**). (**d**–**f**) Alternatively, T cell functionality was assessed using CFSE dilution for ^19^F-PFC-labeled and unlabeled T cells as a result of CD3 crosslinking (**d**) for both CD8^+^ (**e**) and CD4^+^ (**F**) T cells. No significant difference exists in either APC or T cell functionality as a results of ^19^F-PFC labeling (n = 3, mean ± SEM, p > 0.05).

To address APC functionality, a mixed lymphocyte reaction (MLR) was conducted to compare the functionality between unlabeled and ^19^F-PFC-labeled human APC. No significant differences in APC functionality were observed as a result of ^19^F-PFC labeling (Fig. 2a) with respect to stimulating allogeneic CD8^+^ T cell (Fig. 2b) and CD4^+^ T cell (Fig. 2c) proliferation using CFSE dilution as a readout, regardless of the APC to T cell ratio.

To compare the functionality of unlabeled- and ^19^F-PFC-labeled T cells, a T cell proliferation assay assessed their proliferative capacity. Using anti-CD3 coated plates, no difference in T cell proliferation was measured for both CD4^+^ and CD8^+^ T cells (Fig. 2d) using CFSE dilution as a readout (Fig. 2e,f).

### ^19^F cellular MRI *in vivo* signal is the result of originally injected human PBMC

**Figure 3:**
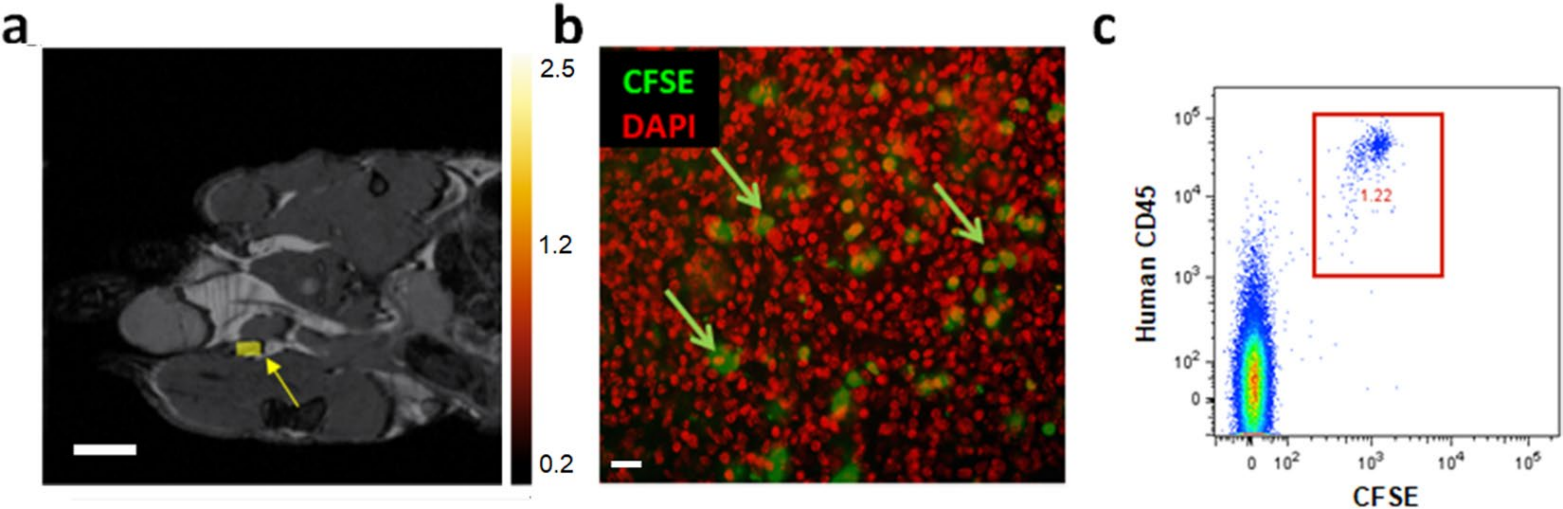
Human CFSE^+^CD45^+ 19^F-PFC-labeled PBMC are present in the popliteal lymph node containing a positive ^19^F MRI signal. (**a**) Two days after the injection of 3 × 10^6^ human ^19^F-PFC-labeled PBMC into the footpad of nu/nu mice, mice were subjected to ^19^F MRI and positive ^19^F MRI signal detection of 1.31 ± 0.2 × 10^5^ cells (yellow arrow) resided within the popliteal lymph node (N = 3, n = 4/experiment; scale bar = 5 mm). (**b**) Following MRI, the lymph nodes were removed, cryopreserved, sectioned and fluorescent microscopy revealed the presence of CFSE^+ 19^F-PFC-labeled human PBMC (representative of n = 8 that did not undergo MRI). The  sections were counterstained with DAPI to reveal nuclei (pseudocoloured red; 100x magnification, scale bar = 10 μm). (**c**) In a similar experiment, draining popliteal lymph nodes were removed and single cell suspensions prepared and counterstained with an antibody specific for human CD45. Flow cytometry analysis revealed the presence of CFSE^+^ human CD45^+ 19^F-PFC-labeled PBMC (**c**, representative N = 3, n = 4/experiment).

Experiments were performed with human PBMC labeled with both ^19^F-PFC and CFSE prior to injection into nude mice in order to verify that the *in vivo* ^19^F signal is the result of originally injected cells. Two days following the injection of 3 × 10^6^ human ^19^F-PFC PBMC into the footpad of nu/nu mice (N = 3, n = 4 per experiment), ^19^F MRI detected 1.31 ± 0.2 × 10^5^ cells (Fig. 3a, yellow arrow) in the draining popliteal lymph node. Following MR imaging, CFSE^+^ human ^19^F-PFC-labeled PBMC were detected in cryosections of the same popliteal lymph node (counterstained with DAPI; Fig. 3b, green arrows). In a similar experiment, two days after the injection of 3 × 10^6 19^F-PFC-labeled CFSE^+^ human PBMC, draining popliteal lymph nodes were removed, single cell suspensions prepared and subsequently counterstained with an antibody specific for human CD45. Flow cytometry identified CFSE^+^ human CD45^+^ cells (0.8778% ± 0.176%; N = 3, n = 4 per experiment) in the popliteal lymph node (representative example shown in Fig. 3c). These experiments confirmed that the signal observed *in vivo* was a result of originally injected human ^19^F-PFC-labeled CFSE^+^ PBMC.

### Safety and viability testing of ^19^F-PFC labeled versus unlabeled PBMC

**Figure 4:**
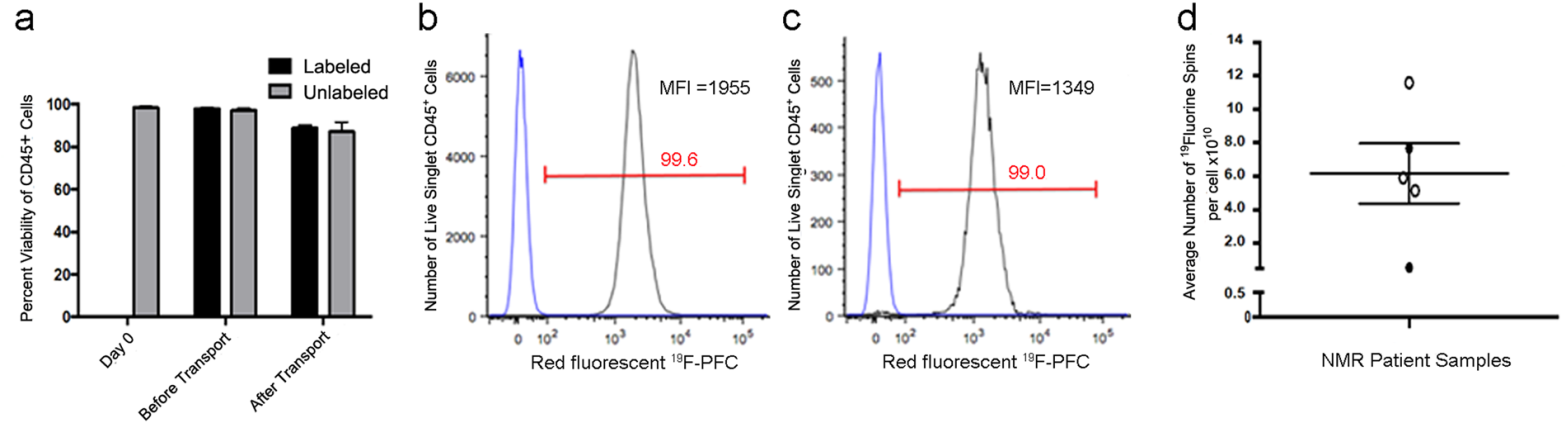
Human PBMC label efficiently with a ^19^F-PFC cell-labeling agent under GMP-compliant conditions and transported without significant loss of viability. (**a**) Following overnight culturing of PBMC with and without the ^19^F-PFC cell-labeling agent (5 mg/mL) under GMP-compliant conditions, labeled and unlabeled PBMC were transported back to London, Canada. The viability of ^19^F-PFC-labeled PBMC and unlabeled PBMC was determined using 7-AAD staining before and after transport. There was no significant decrease in viability because of transport (approximately 2.5 hours for a 250 km distance) as well as no significant difference in viability between labeled and unlabeled cells. (**b**) PBMC were labeled overnight with the red fluorescent version of the ^19^F-PFC cell-labeling agent. Half of the ^19^F-PFC labeled cells were stained for CD45 and for viability and analyzed by flow cytometry which revealed that 99.8% of PBMC were positively labeled with the red fluorescent version of the ^19^F-PFC agent compared to unlabeled PBMC from the same donor (blue histogram). (**c**) The second of half of the labeled PBMC were processed and stained for CD45 and viability ~3 hours later followed by flow cytometry which revealed that 99% of the PBMC retained the red fluorescent ^19^F-PFC compared to unlabeled PBMC from the same donor (blue histogram). (**d**) In order to quantify *in vivo* cell migration, a quantitative assessment of ^19^F-PFC incorporation must also be ascertained using NMR spectroscopy on a known number of PBMC. For the 5 participants analyzed, an average of 6.17 × 10^10 19^F spins was incorporated per cell. For the last three participants, an average of 7.55 × 10^10 19^F spins were incorporated per cell and are denoted by the white circles and are the participants for which MR image data is provided.

We evaluated the quality control and safety testing required for investigational, autologous use of ^19^F-PFC-labeled PBMC in humans. The cell manufacturing facility performed all the PBMC processing and labeling while operating under GMP-compliant conditions as outlined in Supplementary Fig. S1. For all five samples, assessments were conducted for safety (by rapid Gram staining, culture expanded sterility, and mycoplasma testing), purity and identity (presence of >80% CD45^+^ cells), and impurities (absence of endotoxin) and all results met previously outlined specifications (Table 1). We also assessed the viability pre- and post-transport, as assessed by 7-AAD staining. Viability assessment post-transport (<60%) of the first two participants revealed that the transportation conditions were sub-optimal and below the generally acceptable limit (70%) observed by Health Canada. The insipient volume the cells were shipped in was increased to 15 mL in a tube filled with Plasma-Lyte A solution (participants 3–5). With this change in place, the viability of the next three participant samples post-transport for ^19^F-PFC-labeled and unlabeled conditions, respectively, was 88.75 ± 1.55% and 87.1 ± 4.57% (Fig. 4a). However, there was a small but non-significant decrease in viability noted between pre- and post-transport between the cell manufacturing facility and Robarts Research Institute (Fig. 4a). Thus, when appropriate transport protocols were applied the cell viability of the labeled PBMC arrived suitable for autologous injection into humans.

**Table 1:** Cell products from all participants passed all requirements outlined by Health Canada to allow for injection into humans upon clinical trial approval.

	Participant 1		Participant 2		Participant 3		Participant 4		Participant 5	
	Unlabeled	Labeled		Labeled	Unlabeled	Labeled	Unlabeled	Labeled	Unlabeled	Labeled
Stat Gram Stain	Pass	Pass	Pass	Pass	Pass	Pass	Pass	Pass	Pass	Pass
Endotoxin	Pass	Pass	Pass	Pass	Pass	Pass	Pass	Pass	Pass	Pass
CD45+	99.3%	99.7%	84.4%	92.5%	78.9%	96.0%	94.7%	97.1%	98.4%	98.2%
14-day Sterility	Pass	Pass	Pass	Pass	Pass	Pass	Pass	Pass	Pass	Pass
Mycoplasma	Pass	Pass	Pass	Pass	Pass	Pass	Pass	Pass	Pass	Pass

### Human PBMC can be efficiently labeled with a ^19^F-PFC cell labeling agent under GMP-compliant conditions

We next determined that ^19^F-PFC labeling via a GMP-compliant protocol, as performed at the cell manufacturing facility, was designed properly for optimal labeling. Using the fluorescent version of a ^19^F-PFC-labeling agent an aliquot of PBMC was labeled overnight (~20 hr). The shift in mean fluorescence intensity of the fluorescently tagged ^19^F-PFC indicated that 99.8% of the viable singlet PBMC were labeled indicating that all cell lineages contained within PBMC were labeled. In a separate set of experiments (N = 3 different PBMC donors) labeling was conducted as described above but after overnight labeling with the fluorescent version of the ^19^F-PCF, half the cells were assessed immediately for ^19^F-PFC uptake, while the other half was processed as indicated in Supplementary Fig. S1 and left at room temperature to simulate the combined 2.5 hr transport time and post-arrival processing time. Flow cytometry revealed that 98.1 ± 1.2% of PBMC were labeled with the fluorescent ^19^F-PFC overnight (Fig. 4b) and that the percentage of labeled cells remained unchanged (98.3 ± 0.76%, p = 0.87) ~3 hr later (Fig. 4c). There was no significant difference (p = 0.43) in the median fluorescence intensity between the pre- and post-transport samples for the three donors. A quantitative assessment was conducted to determine the average number of ^19^F spins per cell for each participant sample using NMR spectroscopy (Fig. 4d). For all 5 participants who underwent NMR analysis, loading values ranged from 5.14 × 10^10^ to 1.16 × 10^11 19^F spins per cell. For the last 3 subject samples (viability was >87%) an average loading of 7.55 × 10^10 19^F spins per cell was calculated.

### ^19^F-PFC labeling does not alter the PBMC cell lineage composition

Each cell lineage within the PBMC mixture serves a different immunological purpose within a heterogeneous cell-based cancer vaccine. Therefore, the cell lineage composition and phenotype of individual participants’ PBMC preparations were characterized. Both whole blood and PBMC were examined to not only compare labeled and unlabeled PBMC populations, but to also verify that ^19^F-PFC labeling did not significantly alter cell lineage composition when comparing blood drawn on day 0 to PBMC after GMP-compliant processing. Single, live CD45^+ 19^F-PFC labeled and unlabeled PBMC were analyzed (see the flow cytometry gating strategy in Supplementary Fig. S2). The summary of cell lineage phenotyping for the last 3 participants after cell transportation optimization is presented in Table 2. The lymphoid cell lineage and the CD11b^+^CD16^+^ lineage of monocytes, although different between participants, appears to be similar for both labeled and unlabeled conditions within the same participant. However, within the myeloid lineage there was a decrease in the percentage of CD11b^+^CD14^+^ cells recovered post-labelling.

**Table 2:** PBMC cell lineages compared between ^19^F-PFC-labeled (L) and unlabeled PBMC (UL).

Participant Sample Identifier	Surface Antigen (Percentage of live, singlet, CD45^+^ cells)													
	CD45^+^		CD45^+^, CD11b^−^, CD3^+^, CD19^+^, CD20^+^		CD45^+^, CD11b^+^, CD3^−^, CD19^−^, CD20^−^		CD45^+^, CD11b^+^, CD3^−^, CD19^−^, CD20^−^, CD16^+^		CD45^+^, CD11b^+^, CD3^−^, CD19^−^, CD20^−^, CD14^+^		CD45^+^, CD11b^+^, CD3^−^, CD19^−^, CD20^−^, CD14^−^, CD16^−^, CD11c^+^		CD45^+^, CD11b^−^, CD3^−^, CD19^−^, CD20^−^, CD14^−^, CD16^−^, CD56^+^	
	**UL**	**L**	**UL**	**L**	**UL**	**L**	**UL**	**L**	**UL**	**L**	**UL**	**L**	**UL**	**L**
Participant 3	78.9	96.0	79.2	83.7	15.6	6.90	10.7	3.95	3.12	0.36	1.52	2.60	0.625	1.89
Participant 4	90.5	95.0	83.8	87.7	13.6	8.96	0.22	0.42	9.12	0.53	ND	ND	0.0002	0.08
Participant 5	89.6	93.2	77.2	67.4	19.2	21.9	12.9	17.4	3.56	0.42	7.80	4.52	0.003	7.87

Note: We reported the CD45^+^, CD11b^+^, CD3^−^, CD19^−^, CD20^−^, CD14^−^, CD16^−^, CD11c^**+**^ cell population percentage as percentage of previous gate. ND not determined.

### ^19^F-PFC-labeled human PBMC detected using ^19^F cellular MRI in an immunocompromised mouse model

**Figure 5:**
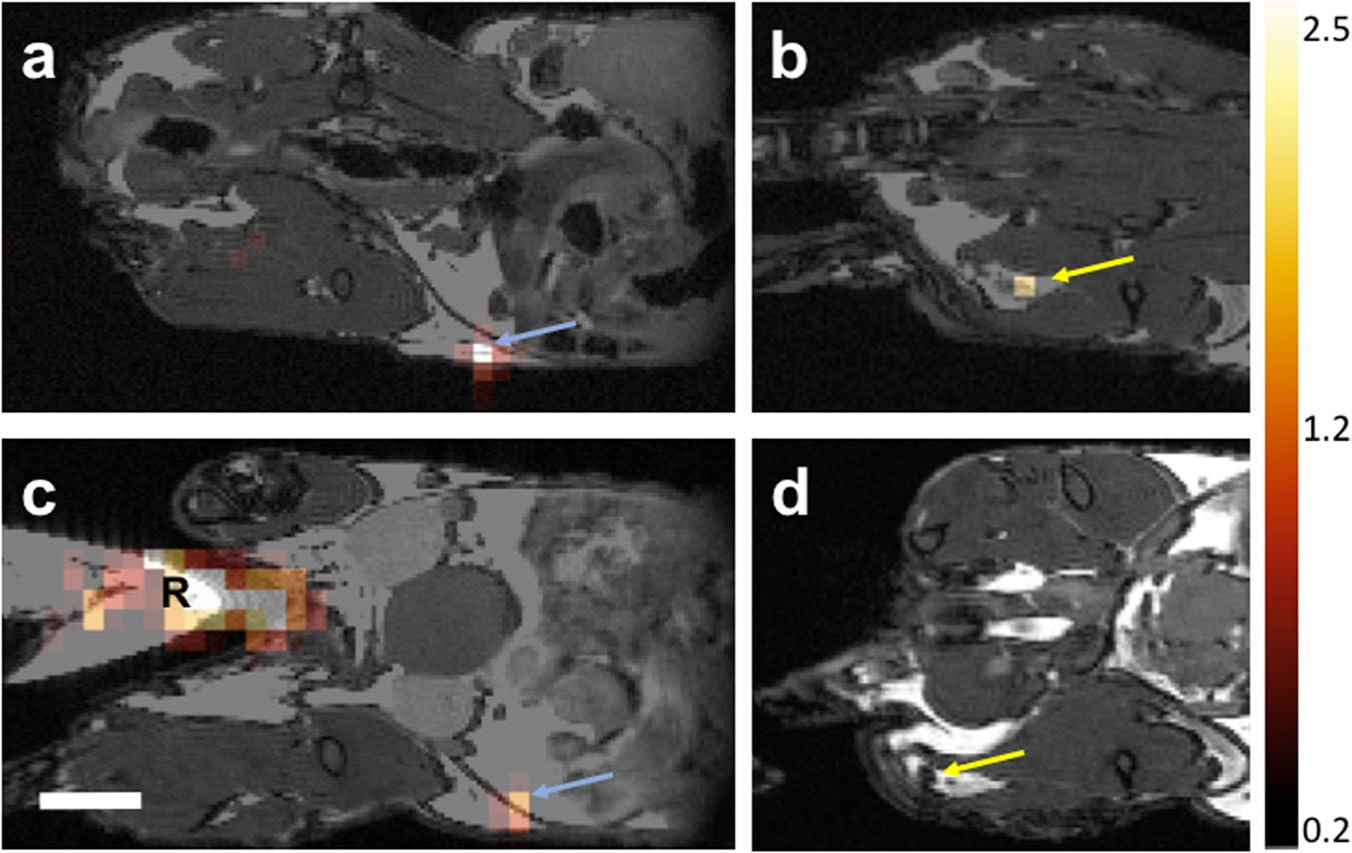
MRI of human PBMC after flank administration in a mouse model. (**a**) Overlay produced from ^19^F and proton MR images following subcutaneous flank injection of 6 × 10^6^ PBMC from participant 4. (**b**) On day 0, a ^19^F signal was observed in both the flank (blue arrow) and at the popliteal lymph node (yellow arrow). (**c**,**d**) Forty-eight hours after administration, the ^19^F signal was decreased but visible in the flank (**c**), but not detectable in the lymph node (**d**). The reference tube used to quantify the number of PBMC is marked with R in panel c. Scale bar = 5 mm.

The number of ^19^F spins determined for each participants’ ^19^F-PFC-labeled PBMC preparation was generally lower (Fig. 4b) than reported for other cell types (10^10^–10^11^ vs 10^12^)^30,33^. To determine if the ^19^F-PFC-labeled PBMC preparations were detectable *in vivo* by cellular MRI, ^19^F-PFC-labeled PBMC from participants 3–5 were injected into the flanks of nude mice. ^19^F-PFC-labeled PBMC were formulated into low and high cell number injection preparations, in which cell numbers varied based on individual participant yield. This information and quantitative ^19^F-MRI results are summarized in Table 3. MR images were obtained at ~2 hours and 2 days after injection. No ^19^F signal was detected at the unlabeled PBMC contralateral flank injection sites or in the draining lymph nodes. For the two mice injected with 1 × 10^6^ and 6 × 10^6^ labeled PBMC from participant 4 (healthy control) MR imaging detected 6 ± 0.3 × 10^5^ and 5.2 ± 0.2 × 10^6^ (Fig. 5a, blue arrow) ^19^F-PFC-labeled PBMC at the injection sites on day 0. On day 2, these numbers were reduced at the low and high injection site to 2 ± 0.3 × 10^5^ and 4.11 ± 0.2 × 10^6^ PBMC, respectively (Fig. 5c, blue arrow). The ^19^F-PFC-labeled PBMC were also detected in the popliteal lymph node in the high dose (6 × 10^6^ labeled PBMC) injected mouse at 2 hours post-injection at day 0 and was quantified to be 2 ± 0.3 × 10^5^ PBMC (Fig. 5b). However, no popliteal lymph node signal was observed 2 days post-injection (Fig. 5d). For mice injected with labeled PBMC from participants 3 and 5 (both prostate cancer patients) ^19^F signal was detected only after the high cell dose injection at both day 0 and 2 but not in any of the draining lymph nodes. Following high dose injection of 5.7 ± 0.1 × 10^6^ labeled PBMC from participant 3 1.13 ± 0.3 × 10^6^ and 7.5 ± 0.3 × 10^5^ PBMC were detected at day 0 and day 2, respectively. With respect to participant 5, the high dose injection of 4 × 10^6^ labeled PBMC permitted for the detection of 1.11 ± 0.2 × 10^6^ PBMC at the injection site on day 0, and 5.9 ± 1.6 × 10^4^ PBMC on day 2. The latter was the lowest cell number detected.

**Table 3:** ^19^F-PFC labeled PBMC detection by cellular MRI following injection in immunocompromised mice.

	Participant 3		Participant 4		Participant 5	
**Injection Cell Number** Subcutaneous Flank	**1 × 10** ^**6**^	**5.7 × 10** ^**6**^	**1 × 10** ^**6**^	**6 × 10** ^**6**^	**1 × 10** ^**6**^	**4 × 10** ^**6**^
**Day 0 Detection** Injection Site^a^	**No**	**Yes** (1.13 × 10^6^)	**Yes** (6 × 10^5^)	**Yes**^**b**^ (5.21 × 10^6^)	**No**	**Yes** (1.11 × 10^6^)
**Day 2 Detection** Injection Site	**No**	**Yes** (7 × 10^5^)	**Yes** (2.0 × 10^5^)	**Yes** (4.11 × 10^6^)	**No**	**Yes** (5.9 × 10^4^)

^a^Day 0 scan completed ~2 hours post injection.

^b^Day 0 scan also detected 2 ×10^5^ cells in the popliteal lymph node.

### MR Imaging of human PBMC under clinical conditions at 3 T

**Figure 6:**
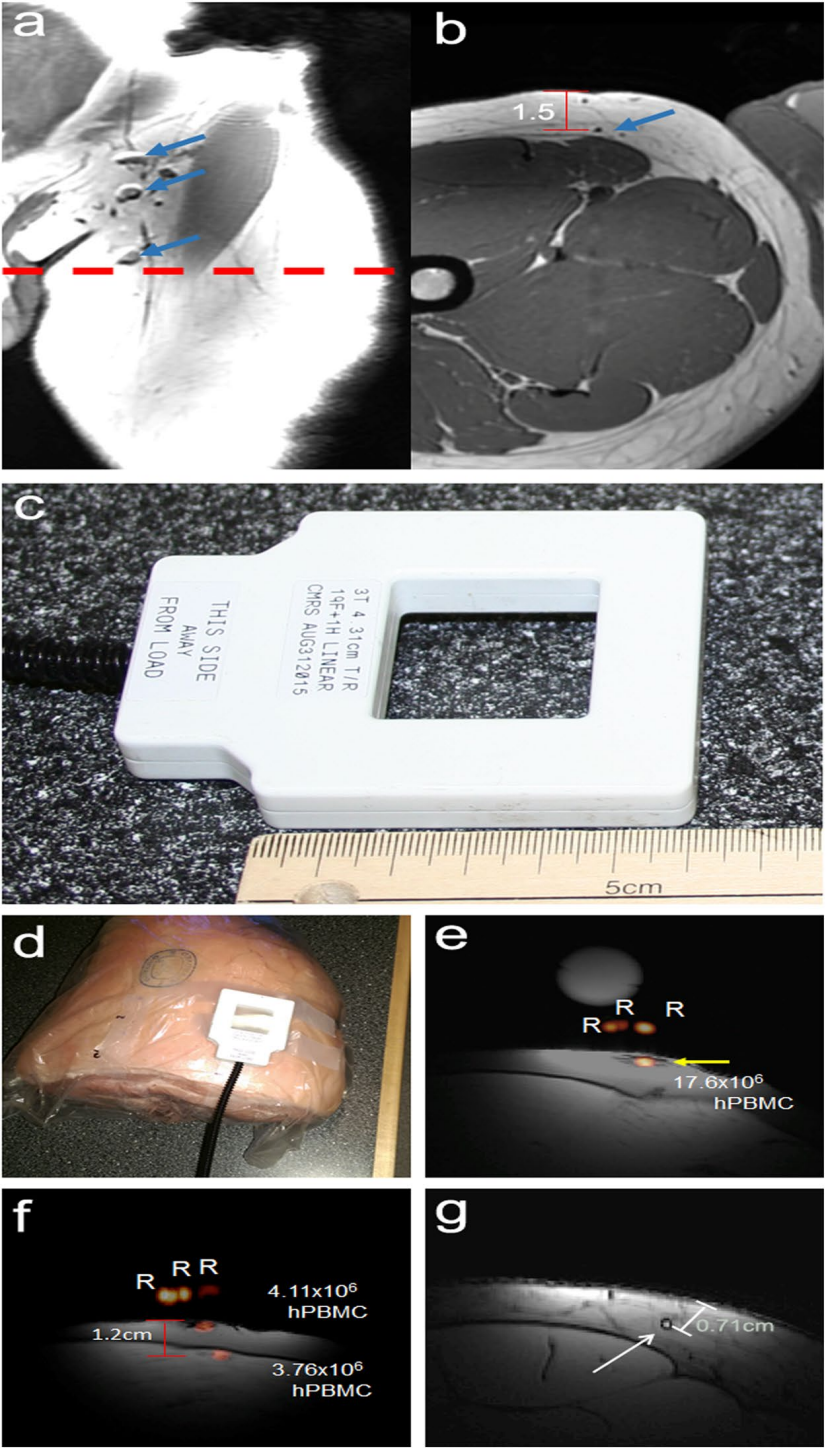
A clinical 3 T MRI scanner detects ^19^F-PFC-labeled human PBMC. (**a**) A T1-weighted, coronal orientation MR image obtained using a body coil reveals human inguinal lymph nodes as dark spheres (blue arrows) within the fat of the upper thigh. The red dashed line indicates the location of the axial MRI slice (**b**). In the axial image, the position of the lymph node is measured to be approximately 1.5 cm below the skin (**b**, red scale bar). (**c**,**d**,**e**,**f**,**g**) PBMC were obtained from a healthy volunteer and labeled overnight with the ^19^F-PFC cell-labeling agent before injection into a ham shank. Imaging was performed by placing the ^1^H/^19^F switchable RF (**c**) coil on the ham shank as shown (**d**). Cells were administered intradermally with MR images shown for the, 20 × 10^6^ (**e**, yellow arrow) and the 4.5 × 10^6^ (**f**, yellow arrow) intradermal doses. An additional 4.5 × 10^6^ dose was administered intramuscularly (**f**, yellow arrow), which is visible at a 1.2 cm depth as measured from the MR image. A representative human proton image taken with the same parameters closely resembles the ham images in both tissue appearance and subcutaneous fat deposition (**g**). Scale bars represent 6 cm.

Human inguinal lymph nodes from 3 healthy volunteers could be detected as dark spheres within the bright fat pad in the upper thigh. A representative image (Fig. 6a,b) reveals that multiple lymph nodes were detected with an average volume of 390 ± 290 mm^3^ and at an average depth of 1.5 ± 0.3 cm below the skin. These MR images were used to localize the target imaging area, after which the ^1^H/^19^F dual-tuned surface coil (Fig. 6c) was placed at the center of this region. For the volunteers, ^19^F-PFC-labeled human PBMC cell pellet phantoms ranging from 1 × 10^6^–10 × 10^6^ cells were placed on the surface of the thigh to optimize the ^19^F imaging parameters at 3 T (Supplementary. Fig. S3). Under optimal labeling conditions, with 4.2 × 10^11 19^F spins/cell, as few as 1 × 10^6^ PBMC could be detected (Supplementary Fig. 3).

As a proof of concept, human PBMC were injected into a ham shank (of comparable size compared to an average human thigh). The ^1^H/^19^F dual-tuned coil was placed on the surface of the ham shank above the target location, as shown in Fig. 6d. The ^19^F-PFC-labeled human PBMC (1.2 × 10^11 19^F spins/cell) were administered intradermally in 3 doses: 1.5, 4.5, and 20 × 10^6^. The two higher injection doses were detectable and quantifiable (Fig. 6e,f) but not the lowest dose (1.5 × 10^6^ PBMC). The intramuscular injection of 4.5 × 10^6^ PBMC placed 1.2 cm below the intradermal dose was also visible (Fig. 6f). Quantification of the number of cells indicated 4.11 × 10^6^ cells at the site of the 4.5 × 10^6^ subcutaneous cell injection, 17.6 × 10^6^ cells at the site of the 20 × 10^6^ cell subcutaneous injection and 3.76 × 10^6^ cells at the site of the 4.5 × 10^6^ cell intramuscular injection. Human ^1^H images (n = 2) of the inguinal area using the ^1^H/^19^F/ dual-tuned coil showed the location of a lymph node in the target area to be similar to that observed in the ham shank. A representative ^1^H human image shows a lymph node 0.71 cm below the surface (Fig. 6g).

## Discussion

The aim of this investigation was to develop a clinical protocol to label therapeutic cells of a non-homogeneous nature, such as PBMC, with a ^19^F-PFC cell-labeling agent approved for use in humans. Other cell labeling and contrast agents have been used in the past, such as iron-based labeling agents to track therapeutic cells in pre-clinical and clinical models^5,20^. However, in contrast to ^19^F cellular MRI that employs a PFC formulation, such iron-based labeling agents have not been reported to label all human leukocyte subsets, are difficult to quantify and do not always provide unambiguous detection of labeled cells^20^. Previous work revealed that the quantity of APC that migrated to a lymph node is directly proportional to the magnitude of the ensuing immune response^16^. By using ^19^F-PFC cell labeling agents, the quantitative distribution of a PBMC-based immunotherapeutic APC cancer vaccine to secondary lymphoid organs, such as a lymph node, may provide valuable biomarker information predictive of treatment efficacy. The labeling of a single uniform cell population, such as DC that are naturally endocytic, with ^19^F-PFC-based cell labeling agents at levels that support detection by cellular MRI is easily achieved^31,36^. However, labeling non-homogeneous populations of cells, such as PBMC, presents difficulties as each cell type has varying endocytic/phagocytic activity and inherent differences in cell volumes that limit PFC label uptake capacity.

The conditions (cell density, ^19^F-PFC Cell Sense concentration and labeling time) for PBMC labeling were based in part on previously published reports, advice from the manufacturer the ^19^F-PFC, as well as our own dose response experiments^22,30,32,37^. We also had to take into account, when planning the actual human clinical trial, that the labeled PBMC needed to be shipped back such that the injection of the participant with the autologous-labeled PBMC and subsequent MRI scan takes place at a reasonably convenient time of day. Our data demonstrate that the main leukocyte subsets, including those known to function as APC, are labelled with a ^19^F-PFC cell labeling agent to a high percentage using the cell culture conditions that we have defined. Using a fluorescent version of a ^19^F-PFC labeling agent and flow cytometry, 99% of PBMC were found to be labeled after the overnight labeling period. We also determined that 99% of the labeled PBMC retained the ^19^F-PFC over the ~3 hr period it takes to ship the labeled PBMC from the UHN manufacturing site (Toronto, ON) to the Robarts Research Institute in London, ON and process them for flow cytometry. Although the average of the median fluorescence intensity (MFI) for the three donors was not different pre- and post-transport, one donor did exhibit a decrease in MFI. The reason for the apparent decrease in MFI is unclear as the manufacturer claims the ^19^F-PFC is stably retained in viable cells. However, leukocytes are known to produce exosomes^38^ and it is possible that over time a portion of the ^19^F-PFC is lost in released exosomes. Additional experiments are required to resolve this issue. Cell labeling was done without affecting the functionality of the blood-derived APC or T cells within the PBMC mixture. Although the PBMC labeling percentage was near 100%, there is some heterogeneity in the amount of label incorporated into each leukocyte subtype. This is attributable to the different cell lineages within PBMC. For example, smaller cells such as T cells and B cells with a small cytoplasmic volume have been shown to label with ^19^F-PFC labeling agents on the low end of 10^12 19^F spins per cell^39^. Larger leukocytes, such as monocytes and dendritic cells label, on the high end of 10^12^ to the lower end of 10^13 19^F spins per cell^31,40^ (and our unpublished data). Furthermore, the ^19^F-PFC labeling agent incorporates into multilamellar macropinosomes^39,41^, so cells highly endocytic or phagocytic in nature, like dendritic cells and monocytes, accumulate more ^19^F-PFC label in culture. However, the above studies all labeled homogeneous cell populations and thus, did not consider the effect of labeling of individual cell populations within a heterogeneous cell population. This likely explains why loading in these studies is higher than what is observed with our PBMC labeling method, as other preliminary studies in our lab have shown increased labeling of each cell lineage (B cells, T cells, monocytes) following negative selection and culturing each separately as a homogeneous population.

Variability within the number of average ^19^F spins per cell was also observed between participants in this study, ranging from 5.14 × 10^10^ to 1.16 × 10^11 19^F spins per cell. A potential explanation for this is the differences in cell lineage percentages between participants. For example, a patient with a higher percentage of B and T cells and a lower percentage of monocytes would have a lower average loading than a patient with a higher percentage of monocytes in their PBMC. Although speculative, considering the small number of participants in this study, the age and/or health status of the donor may also be a factor as intracellular loading was highest within a younger, healthy control (participant 4) compared to older (60–90 years of age) prostate cancer patients (participants 1, 2, 3 and 5). However, a more extensive study is needed to verify this observation.

After establishing ^19^F labeling protocols for PBMC, it was necessary to logistically design a GMP-compliant processing protocol. This protocol involves drawing blood from patients in one location (London Regional Cancer Program, London, Ontario), transporting it to a GMP-compliant cell manufacturing facility (UHN, Toronto, Ontario) 2.5 hours (250 km) away and then returning the blood to Robarts Research Institute (London, Ontario) the next day in a manner suitable for use in an actual clinical trial. Furthermore, this study was designed to have cell processing pass all requirements outlined by Health Canada for autologous injection back into humans^42^. All tests results passed Health Canada’s requirements, except for the PBMC viability of participants 1 and 2. In the case of participants 1 and 2, shipment of PBMC samples back to the Robarts Research Institute post-labeling were sent in small pre-determined volumes in individual vials to be used directly for injection or for further safety testing. Thus, ^19^F-PFC-labeled PBMC recovery, in particular, was low and the viability fell below requirements for injection into humans. While Participant 2 (prostate cancer patient) had a low lymphocyte count (predominant cell type in PBMC) and a low yield, the yield did not seem to be necessarily dependent on lower immune cell counts as participant 3 (prostate cancer patient) had a good yield but a low immune cell count. We rectified the yield problem by subsequently shipping all the PBMC in one 15 mL polypropylene Falcon tube fully filled with Plasma-Lyte A solution. This change alone greatly increased the viability of samples post-transport to levels acceptable to Health Canada. PBMC viability was also not significantly affected by ^19^F-PFC labeling when compared to unlabeled PBMC and was unaffected because of shipment time, distance traveled and the outside temperature. The viability following ^19^F-PFC labeling is similar to what has been reported in other studies using DC and T cells^29,32^. This is an important concept with respect to cell labeling agents and cell-based cancer vaccines as their purpose is to allow for *in vivo* detection of cell migration without having any effects on viability or phenotype that could alter the immunogenic potential of the vaccine.

With heterogeneous cell-based vaccines, each cell lineage within the PBMC mixture serves a different immunological purpose^43–45^. Therefore, it was of interest to determine whether ^19^F-PFC labeling of PBMC affected any cell lineage within the PBMC formulation. Our flow cytometry analysis revealed that, although differences in percentages of lymphocytes and CD11b^+^CD16^+^ monocytes occurred between participants, the percentages between labeled and unlabeled PBMC for each participant was similar. We did note a decrease in the CD11b^+^CD14^+^ monocyte population post-labeling. We have observed that PFC labeling tends to increase the propensity of monocytes to adhere to plastic even when highly hydrophobic, non-adherent plastic was used, thereby resulting in a reduction of this cell type in the final cell suspension and not necessarily indicating CD11b^+^CD14^+^ cell death as a result of labeling. Alternatively, CD14 receptor internalization may have resulted from uptake of the ^19^F-PFC nanoparticle emulsion, as has been suggested for CD36 on murine dendritic cells following iron oxide-based labeling agent phagocytosis and CD14-mediated macropinocytosis^46,47^. This could account for a transient decrease in CD14 on the cell surface of these monocytes. CD56^+^ NK cells exhibited differences between labeled and unlabeled populations in each of the 3 participants PBMC assessed. The reason behind the decrease in the unlabeled population is not clear but may have to do with the lack of proper cytokine support needed for maintaining cultured NK cells. Although broad cell lineage composition was assessed in this study, a detailed phenotypic assessment of individual leukocyte subsets was not assessed.

Pre-clinical *in vivo* imaging studies in mice were conducted to determine the sensitivity for detection of ^19^F-PFC labeled PBMC and development of protocols in pre-clinical models prior to application in human studies. In a future clinical trial, autologous PBMC injection into patients is expected to follow the same processing and set-up protocols as the *in vivo* mouse imaging studies presented here. In all sites receiving ^19^F-PFC-labeled PBMC high dose injections and for one low injection (participant 4), ^19^F-PFC labeled cells were detected on day 0. These are cells that have either not migrated in the 2 hour time frame following injection or could also represent a dead cell population as a result of shearing during expulsion through the 28G1/2 syringe needle. This will be less of factor in the corresponding human PBMC injections as the needle gage will be increased. Quantification at the site of injection 2 days later was always possible if day 0 detection was present, albeit at a lower cell number. This can be attributed partially to cells migrating away from an area of quantified signal to a distal site and partially to dying/dead cells releasing the ^19^F-PFC which will rapidly dissipate or be phagocytosed by mouse macrophages^37^ and cleared from the area of signal quantification. Only live cells retain the ^19^F-PFC^33^. In addition, migration from the flank injection site to a draining popliteal lymph node (quantified to be 2 × 10^5^ cells) was only observed once in one animal. This suggested that, although a small drop in viability occurs as a result of labeling and transport, ^19^F-PFC-labeled PBMC were still able to migrate to biologically relevant sites after injection, which strengthens the case that labeling does not affect the *in vivo* competency of PBMC. Migration to the lymph nodes likely occurred in additional cases, but in insufficient numbers to reach the threshold needed for ^19^F cellular MRI detection. We did not activate the PBMC in any way to stimulate them to migrate to and, in particular, to be retained in secondary lymphoid tissues as has been done for the PBMC-based immunotherapy Provenge using granulocyte-macrophage colony stimulating factor^48^. This may also have reduced the numbers of detectable PBMC in any given draining lymph node. Furthermore, the injections were done in the flank area which can drain to multiple lymph nodes. This is in contrast to what we showed *in vivo* for DC^49^ and, as shown here for PBMC, in which migration from footpad to popliteal lymph is observed as it is the only lymph node that drains immediately from the footpad. However, no migration to other more distant secondary lymphoid tissues was detected by cellular MRI. This may be a limitation of the mouse model in which the number of leukocytes injected is much lower than what can be done in humans^48,50^.

The translational potential of ^19^F-cellular MRI was investigated with a clinical 3 T MRI scanner. A fresh ham shank was employed as a mock human leg due to the strong tissue similarities. The largest factor governing successful detection of PBMC was the number of ^19^F spins/cell. Mean intracellular loading of PBMC ranged from 10^10^–10^11^ between participants, representing an order of magnitude difference in cell detection threshold (10^7^ vs 10^6^ PBMC, respectively). While in the developmental stages of clinical ^19^F-MRI, it may be necessary to pre-screen patients based on intracellular loading. In comparison to other clinical ^19^F studies, PBMC were observed to label on average an order of magnitude lower than dendritic cells (3.9 × 10^12 19^F spins/cell)^30^ and stromal vascular fraction cells (SVF) (2.8 ± 2 × 10^12 19^F spins/cell)^33^. This translates into a proportional decrease in signal. Yet, despite this inherent drawback, the minimum number of detectable cells was comparable to previous studies (DC: ~5 × 10^6^, SVF: 2 × 10^6^, PBMC: 4.5 × 10^6^)^32,33^. The clinical protocol and hardware used when imaging the ham shank represents a 10 times improvement in sensitivity compared to previously reported studies. This improvement is largely due to the small size and high sensitivity of the surface coil used, as well as the higher number of imaging averages employed compared to similar studies^32,33^. To provide higher detection sensitivity, we chose an MRI RF surface coil, but at the cost of limiting the imaging field of view compared to a volume coil. However, due to the superficial location of the proposed injection sites and shallow depth of the target lymph nodes, imaging at depths greater than 1.5 cm is currently not necessary. Improvements in acquisition with different MRI sequences, such as ultrashort echo time (UTE); advanced array coil configurations; post processing techniques, such as compressed sensing; and novel ^19^F labeling agents^27^ will be required to increase sensitivity further.

In this study, we present the highest sensitivity for ^19^F detection reported thus far in the literature using a clinical scanner and clinical protocol. Moreover, we demonstrate it is possible to quantify the signal of ^19^F-PFC-labeled PBMC at a depth of 1.2 cm using a clinical protocol and ^19^F MRI scanner hardware, as the only other study has detected signal of surface injected cells^32^. This type of imaging is valuable when considering that it can be used as a non-invasive technique to track their anatomical location, their persistence and potentially lead to improvements in APC-based cancer vaccine efficacy. This imaging technique can potentially be applied to other types of cell-based cancer therapies, such as chimeric antigen receptor (CAR) T or NK cell immunotherapies. Furthermore, the *in vivo* cell tracking of immunosuppressive T regulatory cell-based therapies to their intended target locations for the treatment of autoimmune disease and organ transplantation is an unmet need required to verify the mechanism behind such immunosuppressive therapies^51,52^. Immunomodulatory mesenchymal stem cell therapy trials also have a similar requirement^33^. Regulatory agencies are considering whether to require cell tracking to establish the anatomical fate(s) and persistence of the therapeutic cells as part of the process of establishing treatment efficacy. Thus, it is important to have a firm understanding of the advantages and disadvantages of different cell labeling agents such ^19^F-PFC formulations.

## Methods

### Mice

Male nu/nu mice (8–10 weeks) were purchased from Charles River Laboratories Inc. The Animal Care Committee at Western University pre-approved all animal experiment reported here (Protocol #2015-046) in accordance with the Canadian Council for Animal Care guidelines. All experiments involving animals were performed in accordance with relevant institutional, provincial and national guidelines and regulations.

### Participants

The University of Western Ontario Health Sciences Research Ethics Board (REB) pre-approved this protocol (REB Protocol #104593). Each participant involved in this study protocol received a REB-approved Letter of Information and provided a signed Letter of Informed Consent before entering into the study. For inclusion in the study, healthy participants had to be 18 years of age or older and free of medical conditions that precluded participation in the study. Potential participants who were undergoing chemotherapy or radiation therapy, undergoing treatment with an immunosuppressive drug (*eg*. steroid) or infected with Human Immunodeficiency Virus, Hepatitis B or C Virus or other transmissible diseases were excluded from the study. Participants with prostate cancer were required to have castrate resistant, non-metastatic prostate cancer with normal or rising prostate specific antigen (PSA) levels. Prostate cancer participants had to be otherwise healthy and free of other serious medical conditions. Prostate cancer patients on hormone therapy were permitted to participate but were excluded from the study if they were undergoing active chemotherapy or radiation therapy, being treated with immunosuppressive drugs (*eg*. steroids), were infected with Human Immunodeficiency Virus, Hepatitis B or C Virus or other transmissible disease.

### GMP-compliant human peripheral blood mononuclear cell isolation

Following venopuncure, all experimental procedures involving human blood or the derived labeled cell products were performed in accordance with the Ontario Health and Safety Act and in laboratories pre-approved by the University of Western Ontario’s or the University of Toronto’s Biosafety/Biohazards Committee for Containment Level 2 work as mandated by Health Canada’s Canadian Biosafety Standards and Guidelines (2013). All personnel used personal protective equipment and handled all blood products as if they are contaminated to exercise the highest degree of caution as mandated by Health Canada’s Canadian Biosafety Standards and Guidelines (2013). PBMC were isolated from 100–160 mL of blood from a healthy volunteer (n = 1, participant 4) and prostate cancer patients (n = 4; participants 1, 2, 3, 5). The blood was drawn into EDTA vacutainer tubes and transported in a container that maintained a temperature of 20–25 °C for 2–3 hours from London, Ontario to the University Health Network (UHN, Toronto, Ontario) cell manufacturing facility. All subsequent steps of the GMP-compliant manufacturing process were conducted at the UHN location. Upon receipt, PBMC were isolated by density gradient centrifugation using Ficoll-Paque™ PREMIUM (GMP-grade, GE Healthcare Bio-Sciences).

### Human Peripheral Blood Mononuclear Cell ^19^F-PFC Labeling

Fifty million PBMC were labeled with the ^19^F-PFC (clinical use grade CS-1000; Celsense Inc.) in 75 cm^2^ ultra low attachment flasks (Corning) in defined serum-free AIM V® Medium CTS™ media (GMP-grade, ThermoFisher Scientific) for 20 hours at 37 °C, 5% CO_2_. The ^19^F-PFC-labeled PBMC were collected by gentle scraping and washed with PBS 3 times and resuspended in 15 ml of Plasma-Lyte A (USP grade and GMP compliant, Baxter Canada) for shipment to the Robarts Research Institute (London, ON).

### Cell Viability

A trypan blue exclusion assay monitored cell viability during the process of PBMC isolation. The injection-ready ^19^F-PFC -labeled PBMC viability was performed more rigorously by 7-AAD staining and flow cytometric analysis on a LSRII analytical flow cytometer (BD Biosciences). Alternatively, an aliquot of both ^19^F-PFC-labeled and unlabeled PBMC in HBSS + 0.1% BSA were first stained on ice for 25 minutes with anti-human CD45-FITC, then washed and resuspended in buffer supplied in the Apoptosis Detection kit (Biolegend) at room temperature. Data was acquired immediately after addition of 7-AAD to the PBMC.

### Cell Safety

The following safety tests were performed on each PBMC preparation. The Fourteen-day sterility test was performed on 1 mL of clarified culture supernatant inoculated each into thioglycollate and tryptone Soya tubes. Inoculated tubes were sent to a certified microbiology laboratory (Mt. Sinai Hospital) for analysis as per United States Pharmacopeia (USP) Chapter 71^53^. Endotoxin was measured in 1 mL of clarified cell culture supernatant by a Health Canada approved Endosafe^®^-PTS Portable Test System (Charles River). Samples were processed as per manufacturer recommendations, including a reference standard spike-in (positive control). Mycoplasma testing was done on final ^19^F-PFC-labeled PBMC using a validated qPCR method as per USP Chapter 63^54^. Testing was done on 2–3 × 10^6^ cells/mL collected along with supernatant collected post-harvest but prior to washing.

### Flow Cytometry

The following anti-human antibodies were used for phenotyping: CD45-FITC and CD45-PE (Clone HI30), CD3-PE (HIT3a), CD19-PE (HIB19), CD20-PE (2H7), CD11b-Alexa Fluor 488 (M1/70), CD16-Alexa Fluor 700 (3G8), CD8-PE (SK1), CD4-PerCP (RPA-T4), CD11c-APC (3.9), CD56-BV605 (HCD56), CD14-eFluor450 (61D3) and UltraComp eBeads (Biolegend or eBioscience). To assess uptake of ^19^F-PFC, PBMC were labeled at varying cell (2, 5, 10 × 10^6^ cells/mL) and fluorescent CS-1000 ATM DM Red (Celsense Inc.) ^19^F-PFC (2.5, 5, 7.5 mg/mL) concentrations as above. The following day, labeled PBMC were collected, washed in HBSS and processed for phenotyping as described below. To assess retention of the ^19^F-PBMC, PBMC (at a density of 5 × 10^6^ cells/mL) were labeled with fluorescent CS-1000 ATM DM Red (5 mg/mL) overnight. Following labeling, the cells were washed in HBSS and split into half. The first half was stained immediately for CD45 and viability stain as above and fixed. The second half was resuspended in 15 mL of Plasma-Lyte A and left to for 2.5 hr at room temperature to simulate the shipping time from UHN, Toronto to Robarts Research Institute, London. The cells were pelleted and stained for CD45 and viability and fixed as above. Both halves were then analyzed by flow cytometry.

To phenotype and determine the lineage composition of the PBMC, flow cytometry was performed on whole blood and isolated PBMC samples on the day of blood draw, on ^19^F-PFC-labeled and unlabeled PBMC following overnight culture and upon return to the Robarts Research Institute. Isolated PBMC were blocked on ice for 30 minutes with 5% NGS (v/v) in HBSS + 0.1% BSA and then washed in HBSS and stained with LIVE/DEAD® dye (ThermoFisher) for 20 minutes at room temperature. Following washing in HBSS, cell surface staining with the antibodies mentioned above was conducted in HBSS + 0.1% BSA on ice (4 °C) for 25 minutes. Stained PBMC were washed in cold HBSS and re-suspended in HBSS + 0.1% BSA for data acquisition on a LSRII. Staining of whole blood was performed as described above without a blocking step. Red blood cells were lysed by the addition of ammonium chloride lysis buffer following antibody and LIVE/DEAD® staining. Data was analyzed using FlowJo software (Tree Star).

### Mixed Lymphocyte Reaction (MLR)

PBMC were obtained from a healthy volunteer and using a human CD3 positive selection kit (Stemcell Technologies) T cells were removed from cell suspension. The remaining cells were cultured overnight with or without ^19^F-PFC (5 mg/mL) as described above. Following overnight culture, cells were collected and washed in HBSS and then suspended in AIM V® Medium CTS™ media at 2.5 × 10^6^ cells/mL before serially diluting in triplicate. Then, T cells were enriched from PBMC from different volunteers (n = 3) using a human CD3 negative selection kit (Stemcell Technologies). Isolated T cells were suspended at 1 × 10^6^ cells/mL in warm PBS + 0.5% human AB serum and labeled with CFSE (carboxylfluorescein succinimidyl ester, 1μM) for 10 minutes at 37 °C. The reaction was quenched in ice-cold complete media and washed in HBSS. The T cells were suspended at 3 × 10^6^ cells/mL in AIM V® Medium CTS™ media and 100 μL aliquoted to each well. The MLR was carried out for 4 days at 37 °C/5% CO_2_. After 4 days of incubation, wells were washed in HBSS and the cells stained for CD4 and CD8 as above. CFSE dilution, as determined by flow cytometry, was used as a readout to assess allogeneic T cell proliferation.

### T Cell Proliferation Assay

T cells from healthy volunteers (n = 3) were obtained from PBMC and cultured with or without ^19^F-PFC (5 mg/mL) as described above. The following day, T cells were collected and labeled with CFSE as above. T cells were then suspended at 3 × 10^6^ cells/mL in AIM V® Medium CTS™ media and 100 µL of cell suspension was plated in triplicate. Negative control wells were uncoated while positive control wells were coated overnight with anti-CD3 antibody (2 µg /well in 100 μL PBS).

### Adoptive Transfer of PBMC

Prior to injection, ^19^F-PFC-labeled and unlabeled PBMC were washed twice in PBS and were divided into a low (1 × 10^6^; n = 1/participant) and high (4–6 × 10^6^; n = 1/participant) injection number per 100 µL PBS. Labeled or unlabeled PBMC were injected into the upper left and right flank of anesthetized nude mice, respectively. In some experiments PBMC were labeled with CFSE as described above. Dual-labeled ^19^F-PFC^+^ CFSE^+^ as well as CFSE^+^ control (^19^F-PFC^−^) PBMC were washed and 3 × 10^6^ cell suspended in 40 µL of PBS for injection into the left and right footpad of anesthetized nude mice, respectively. Immediately following ^19^F cellular MRI 48 hours later, draining popliteal lymph nodes were removed and processed as described for histology or for flow cytometry^55^.

### MRI of PBMC Injected Mice

Mouse imaging was performed with a 9.4 Tesla (T) Varian small-animal MRI scanner (located in Containment Level 2 approved room) using a 3D-balanced steady state free precession (bSSFP) sequence that included a means to remove any ^19^F isofluorane signal as previously described^24^. Animals were imaged alongside a reference tube containing 3.33 × 10^16 19^F/μL or 7.3 × 10^16 19^F/μL. Mice were anesthetized with 2% isofluorane. MRI was performed using a dual-tuned birdcage volume coil (diameter 2.2 cm, length 5.1 cm), tuned to 400.2 MHz and 376.8 MHz for ^1^H and ^19^F imaging, respectively. For ^1^H imaging the scan parameters were: repetition time (TR) = 5.0 ms, echo time (TE) = 2.5 ms, receiver bandwidth (rBW) = 78 kHz, flip angle (FA) = 30°, phase cycles (PC) = 4, averages = 3, resolution = 200 × 200 × 200 μm^3^, and number of excitations (NEX) = 3. For ^19^F imaging the parameters were: TR = 4.0 ms, TE = 1.9 ms, rBW = 25 kHz, FA = 70°, PC = 4, averages = 250, resolution = 1 × 1 × 1 mm^3^, and NEX = 200. The total protocol time for both proton and ^19^F imaging was under 90 minutes.

### Clinical MRI Protocol

Optimization was performed with PBMC pellet phantoms produced by centrifugation of 1, 5, and 10 × 10^6^ PBMC in Eppendorf tubes. The cell pellets were overlaid with 1% agarose. Mock human imaging was performed by intradermal injection of 1.5, 4.5, 10.5, and 20 × 10^6^ PBMC into a ham shank in a biosafety cabinent located in a Containment Level 2 approved laboratory. An additional 4.5 × 10^6^ PBMC were administered 1.2 cm below the dermal layer to measure imaging depth sensitivity. The ham shank was first imaged with a GE MR750 (GE Healthcare), 3 T MRI scanner (located in a Containment Level 1 room) equipped with a multinuclear pre-amplifier with the built-in ^1^H body coil to identify regions of interest (ROI). Site-specific imaging was performed by placing a dual ^1^H/^19^F-tuned switchable surface coil (4.3 cm × 4.3 cm; Clinical MR Solutions) directly over the ROI for optimum sensitivity. The dual-tuned surface coil is approved for human use under Investigational Testing Authorization from Health Canada. A 2D fast gradient echo (Fast GRE) sequence was used for ^1^H imaging with the following scan parameters: TE = 2.6 ms, TR = 100 ms, field of view (FOV) = 15 cm × 15 cm × 5 cm, Image Matrix = 256 × 256, Slice thickness = 5 mm, rBW = 83 kHz, NEX = 3, and FA = 20°. Following ^1^H imaging, the coil was switched to ^19^F-mode and the same FOV was scanned. The ^19^F images were obtained with a broad-banded 3D bSSFP based on the GE FIESTA-C sequence. The scan parameters were: TE = 2.2 ms, TR = 4.4 ms, FOV = 15 cm × 15 cm × 5 cm, Image matrix = 46 × 46, Slice thickness = 5 mm, rBW = 10 kHz, NEX = 425 and FA = 70°. ^19^F imaging time was 15 minutes, with a total protocol time under 25 minutes.

### ^19^F-loading Efficiency and Signal Quantification

The mean intracellular ^19^F content of the PBMC was determined by NMR spectroscopy using a 400 MHz Varian vertical spectrometer. A known number of ^19^F-PFC-labeled cells was pelleted, then lysed and transferred to a 5 mm diameter NMR tubes (New Era Enterprises, Inc.) along with 300 μL of D_2_O and 100 μL of 0.1% Trifluoroacetic acid (TFA) as a reference peak for quantifying the number of ^19^F spins/cell. Spectroscopy parameters were TR = 7 s, rBW = 19 kHz, NEX = 100, and spectral range from −68 ppm to −93 ppm. The number of PBMC detected within MR images was determined with Voxel Tracker^TM^ software (Celsense Inc.) as previously described^24,29^.

### Statistical analysis

All data was presented as the mean with the standard error of the mean. A paired T-test (Graph Pad Prism) was used to compare between ^19^F-PFC-labeled and unlabeled PBMC. Significance was considered if p < 0.05.

### Availability of materials and data

We, the authors, agree with the Scientific Reports requirement that we will make materials, data and associated protocols promptly available to readers upon request. The ^19^F-PFC MRI cell-labeling agent, Cell Sense (CS-1000) and the fluorescent version (CS-1000 ATM DM Red) are commercially available from Celsense, Inc.

## Electronic supplementary material

Supplementary Figures S1, S2 and S3
