# Supplementary material for: 19F-perfluorocarbon-labeled human peripheral blood mononuclear cells can be detected in vivo using clinical MRI parameters in a therapeutic cell setting
Source: Sci Rep. 2018 Jan 12;8:590. doi: 10.1038/s41598-017-19031-0 (PMC5766492; doi:10.1038/s41598-017-19031-0)
Supplement: Supplementary file 1 — Supplementary Figures S1, S2 and S3 [file 41598_2017_19031_MOESM1_ESM.pdf]

Supplementary Data for

^{19}F-perfluorocarbon-labeled human peripheral blood mononuclear cells can be detected *in vivo* using clinical MRI parameters in a therapeutic cell setting

Corby Fink, Jeffrey M. Gaudet, Matthew S. Fox, Shashank Bhatt, Sowmya Viswanathan, Michael Smith, Joseph Chin, Paula J. Foster, Gregory A. Dekaban

Supplementary Figure 1. Outline of events for Good Manufacturing Practices (GMP) processing, safety testing and transport of ^{19}F-PFC-labeled PBMC.

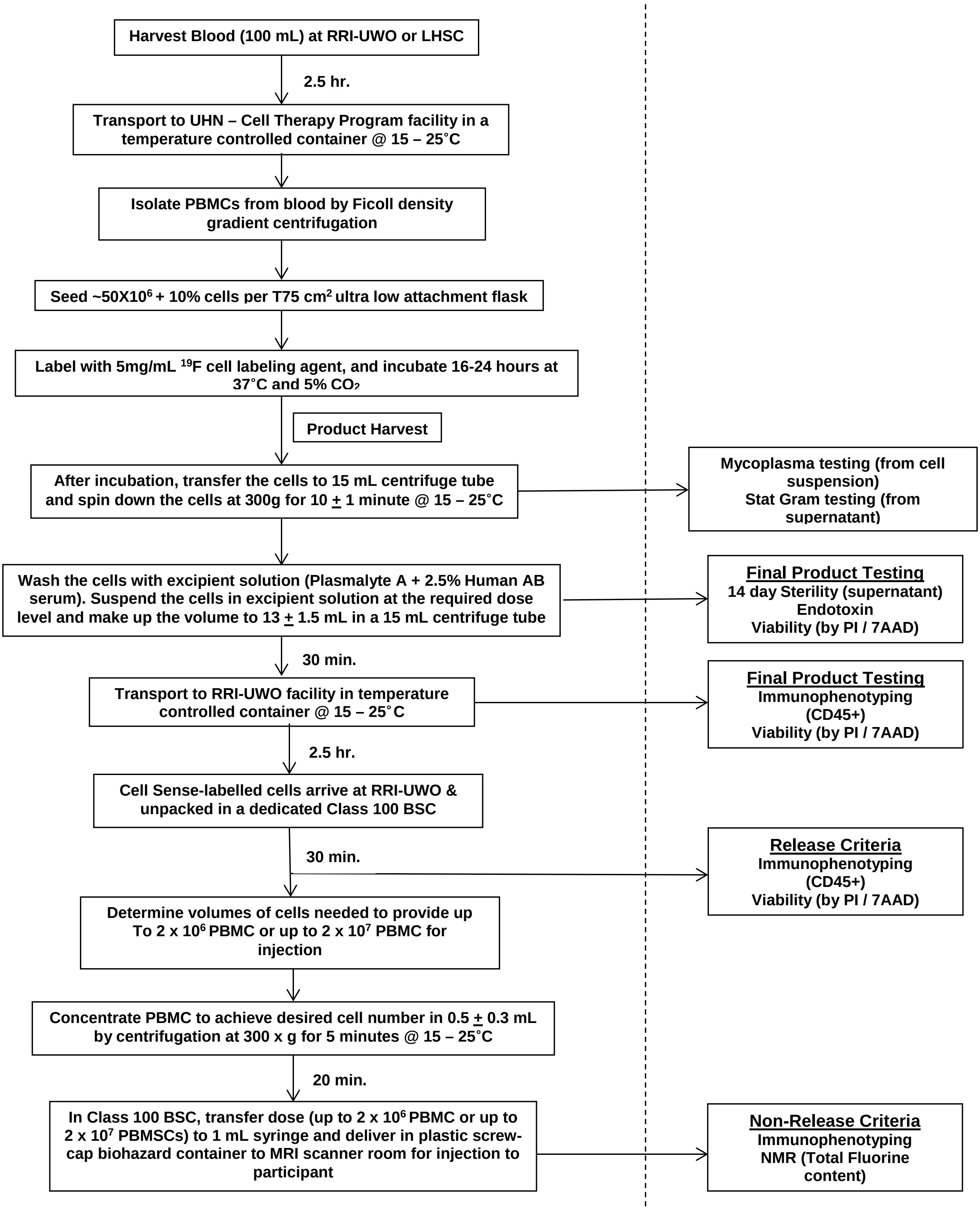
Harvest Blood (100 mL) at RRI-UWO or LHSC
2.5 hr.
Transport to UHN – Cell Therapy Program facility in a temperature controlled container @ 15 – 25˚C
Isolate PBMCs from blood by Ficoll density gradient centrifugation
Seed ~50X10^{6} + 10% cells per T75 cm^{2} ultra low attachment flask
Label with 5mg/mL ^{19}F cell labeling agent, and incubate 16-24 hours at 37˚C and 5% CO_2
Product Harvest
After incubation, transfer the cells to 15 mL centrifuge tube and spin down the cells at 300g for 10 $\pm$ 1 minute @ 15 – 25˚C
Wash the cells with excipient solution (Plasmalyte A + 2.5% Human AB serum). Suspend the cells in excipient solution at the required dose level and make up the volume to 13 $\pm$ 1.5 mL in a 15 mL centrifuge tube
30 min.
Transport to RRI-UWO facility in temperature controlled container @ 15 – 25˚C
2.5 hr.
Cell Sense-labelled cells arrive at RRI-UWO & unpacked in a dedicated Class 100 BSC
30 min.
Determine volumes of cells needed to provide up To 2 x 10^{6} PBMC or up to 2 x 10^{7} PBMC for injection
Concentrate PBMC to achieve desired cell number in 0.5 $\pm$ 0.3 mL by centrifugation at 300 x g for 5 minutes @ 15 – 25˚C
20 min.
In Class 100 BSC, transfer dose (up to 2 x 10^{6} PBMC or up to 2 x 10^{7} PBMSCs) to 1 mL syringe and deliver in plastic screw-cap biohazard container to MRI scanner room for injection to participant
Mycoplasma testing (from cell suspension)
Stat Gram testing (from supernatant)
Final Product Testing
14 day Sterility (supernatant)
Endotoxin
Viability (by PI / 7AAD)
Final Product Testing
Immunophenotyping (CD45+)
Viability (by PI / 7AAD)
Release Criteria
Immunophenotyping (CD45+)
Viability (by PI / 7AAD)
Non-Release Criteria
Immunophenotyping
NMR (Total Fluorine content)

Supplementary Figure 2. Human myeloid and lymphoid cell subsets can be analyzed such that cell lineage percentages can be compared between ^{19}F-PFC labeled and unlabeled PBMC. Blood or isolated PBMC were analyzed for cell lineage composition using the gating strategy described here. Briefly, hematopoetic cells were selected for using CD45^{+} (A), followed by gating on live (B), singlet cells (C). After debris removal (D), the remaining cells were gated on CD11b for myeloid cells or CD3/CD19/CD20 for lymphoid cells (E). For CD11b^{-} and CD3/CD19/CD20^{-}cells, further gating on CD56 can be conducted to identify NK cells (F). For cells that are CD11b^{+}, gating for CD16 and CD14 to analyze monocytes was conducted (F). Lastly, cells that are CD11b^{+} but CD14/CD16^{-} were gated on CD11c to identify dendritic cells (G). All cell surface marker gates were assigned based on their respective fluorescent minus one (FMO) controls.

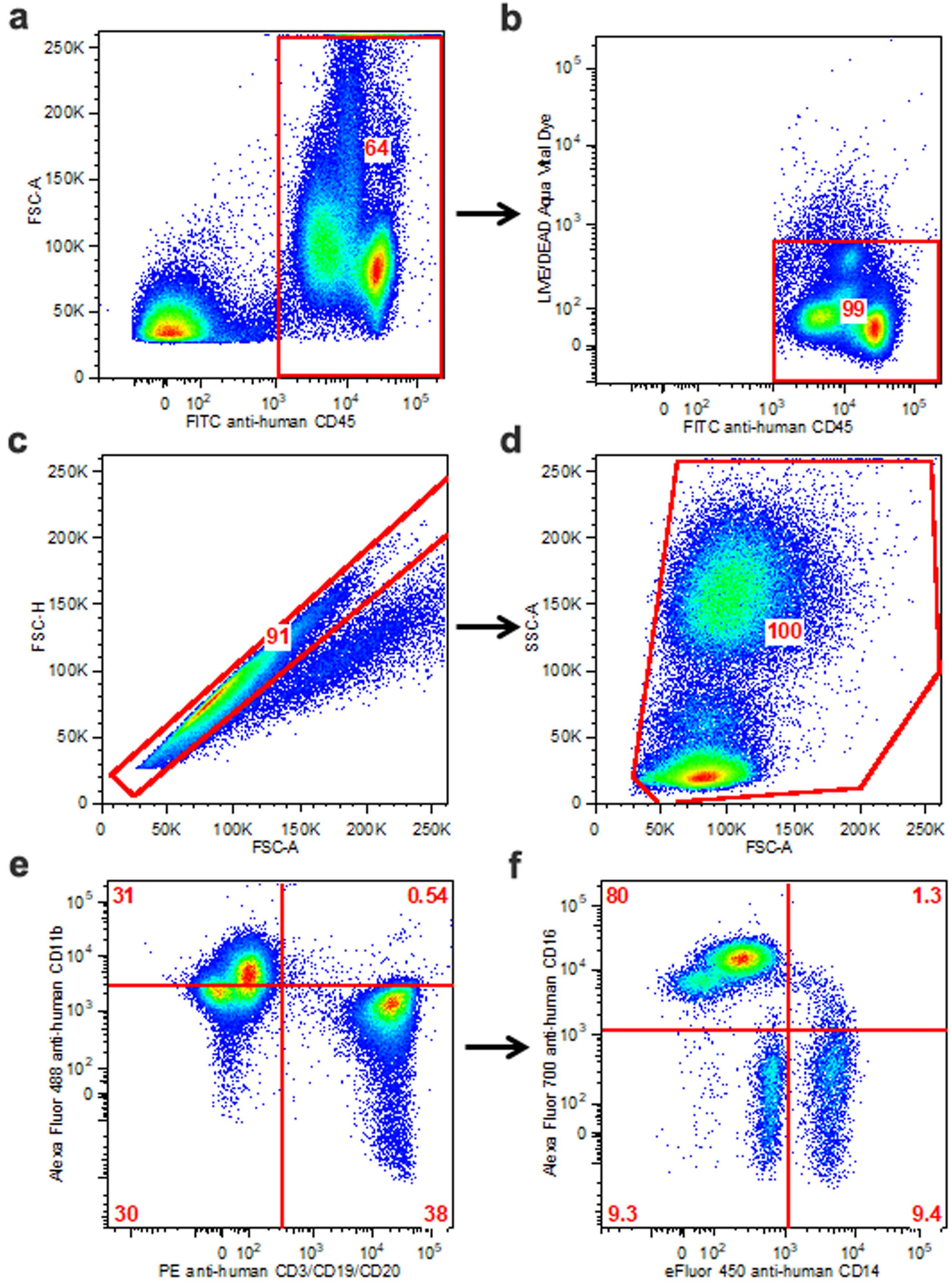

a
b
c
d
e
f
FSC-A
FITC anti-human CD45
64
LIVE/DEAD Aqua Vital Dye
99
FSC-H
91
SSC-A
100
Alexa Fluor 488 anti-human CD11b
PE anti-human CD3/CD19/CD20
31
0.54
30
38
Alexa Fluor 700 anti-human CD16
eFluor 450 anti-human CD14
80
1.3
9.3
9.4

Supplementary Figure 3. Human PBMC pellet phantoms as low as $1x10^6$ can be detected at 3T using a surface coil. PBMC were obtained from a healthy volunteer and labeled with ^{19}F cell labeling agent overnight. Phantoms were created by centrifuging $1x10^6$, $5x10^6$ and $10x10^6$ ^{19}F-labeled PBMC and overlaying with 1% agarose in an eppendorf tube (A). PBMC phantoms were scanned at 3T using a (surface coil) and all 3 phantoms described in (A) were detected using ^{19}F cellular MRI (B), with a hot-iron colour scale used for ^{19}F MRI. Some point spread function artifact is visible from the highest concentration phantom ($10x10^6$ PBMC).

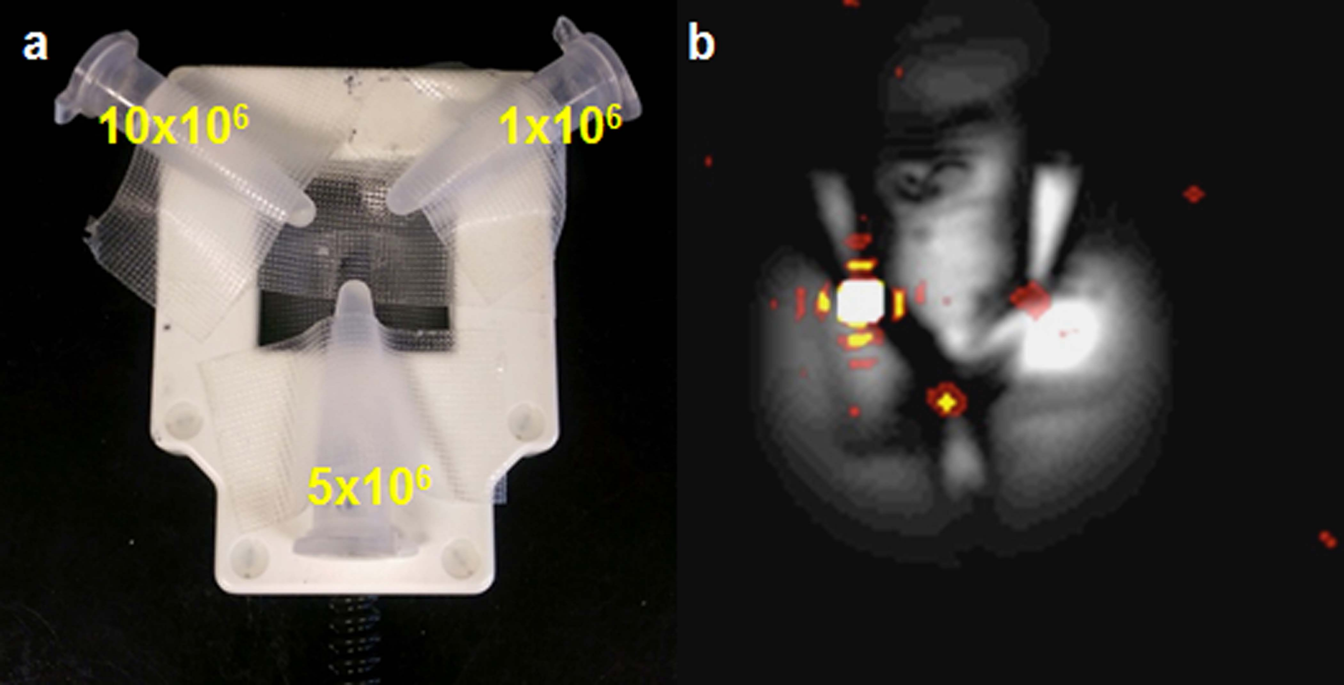
a
10x10^6
1x10^6
5x10^6
b
